# An audit of skills taught in registered nursing preparation programmes in Australia

**DOI:** 10.1186/s12912-015-0113-7

**Published:** 2015-12-17

**Authors:** Roy A. Brown, Patrick A. Crookes, Don Iverson

**Affiliations:** School of Nursing, Faculty of Science, Medicine and Health, University of Wollongong, Wollongong, NSW Australia; Swinburne University, Melbourne, Australia and Visiting Professor, University of Wollongong, Wollongong, NSW Australia

**Keywords:** Skills, Nursing, Eligibility to practice programmes

## Abstract

**Background:**

A competitive Carrick Institute Competitive Grant (CG7-523) was obtained to explore what skills were taught and what assessment of practice approaches were used in nursing programmes in Australia. The intention was twofold; firstly to identify what skills were being taught which would contribute to the development of an assessment of practice toolkit for eligibility to practice programmes in Australia. This paper specifically reports on the skills taught in nursing programmes in Australia.

**Methods:**

A qualitative research methodology was used through a documentary analysis of university curriculum documents. This was undertaken independently by two researchers; the data was then reviewed by an expert group. The skills taught were explored, listed and categorised using a conceptual framework, then refined and reported.

**Results:**

Over 1300 skills were initially identified within nursing programmes across Australia; these were ‘clustered’ using a framework into 30 skills areas. These included psychomotor skills to skills areas that relate to human factors such as communication, team work, leadership and supervision.

**Conclusions:**

A wide range of skills were referred to in university nursing programme curriculae in Australia. There were some significant variations; some universities taught their student nurses how to manage a client/patient requiring external invasive ventilator support. There were however a number of similar skills areas identified; such as acute care assessment skills (monitoring vital signs) and mental health assessment skills. The range of skills taught within nursing curriculum is challenging as there is only limited time to expose students to those skills and afford the student the opportunity to practice those skills in order to achieve competence prior to registration.

## Background

There appears to be a sense of disappointment with the product of contemporary nursing programs in that new RNs (i.e. individuals who have recently completed accredited courses and are eligible for licensure/registration as a nurse) are often referred to as not being ‘competent’ by clinically based colleagues. This has probably been prevalent since the move of nursing education from the hospital based sector to higher education, which began in 1985 and concluded in 1994 [1]. Between 1985 and 1994 nursing curriculum shifted to accommodate the academic structure and accreditation of higher education institutions, as well as state and territory nursing boards; this restructuring of curriculum also led to a reduction in the amount of time that pre-registered nursing students spent in practice. This appears to have led to the idea that the newly registering nurse is therefore ‘less able in practice’.

Since this time, when experienced nurses are asked to indicate what they believe new nurses cannot do that they should be able to do; they often cite a lack of ‘work readiness’ [1]. It appears to be believed that many new nurses cannot be relied upon to perform as a full member of the RN team from day one (i.e. carrying a client/patient load in the typical patient allocation model of care); unreasonable or unrealistic as that may be [2–4]. It is also important to note that typically, these experienced nurse are not referring to the ‘regulatory competencies’ but rather to the individual’s ‘skills set’ or ‘employer competencies’ [5].

Similarly a further common complaint has been that new nurses do not possess adequate time management skills and have difficulty managing the competing priorities of a complex patient load as effectively as (some) experienced colleagues [2–4, 6, 7]. Although time management is often referred to as a ‘basic’ skill, the investigators contend that managing a patient load (particularly if not guided by more senior colleagues) is anything but basic. Rather, time management is complex and requires insights into the many personal and clinical needs of the client/patient group and their relatives, and also the routines and expectations of the clinical area(s) in which the care is being delivered.

Therefore, it may not be surprising that newly registering nurses are seen as lacking in competence basically because expectations are unreasonable [1, 3]. These high expectations could be the result of three major factors: (1) the role of a new RN is not well defined; (2) entry level practitioners are now found in all areas of clinical practice; and, (3) the increased complexity and acuity of care provided. These factors in combination make it virtually impossible for new registrants to ‘arrive in practice’ as competent practitioners. It would be difficult for an experienced RN to be able to do this, let alone a novice one. This is supported by the fact that specialist care delivery units, such as critical care areas [8] currently these standards are under review) and palliative care units [9], have competency frameworks that enable the development of focussed skills in a specific clinical setting that an entry level practitioner may undertake, or an experienced practitioner who is new to that area after moving from another clinical setting. This illustrates that even experienced clinicians should not be expected to move seamlessly between areas of practice.

An important issue that compounds these problems is each University nursing programme in Australia has its own unique curriculum structure and content, including the skills taught and assessed and clinical assessment tool(s) for assessing competence in its’ pre-registration nursing student programmes. Assessment of practice documents are mostly based on the NMBA [10] Competencies and often, between universities, focus on very different nursing skills if, indeed, these skills are overtly indicated or overtly assessed in practice. This suggests that there is scope for different outcomes for registrants both within and between programs. This causes further concern for clinical colleagues because they are often called upon to be involved in the clinical assessment of pre-registration nursing students from a number of universities using a variety of assessment tools.

Nursing practice is a complex interplay of art and science; it is more than a set of individual skills. The nurse requires a range of skills and competencies that when combined is likely to ‘exceed the sum of the parts’. Eraut [11], when discussing professional education, explored technical knowledge and skill acquisition; he indicated that professions give their novices “extensive periods of observation and guided practice” (p:38). Eraut goes onto explore Polyany’s [12] work around ‘tacit’ knowledge; this is the ‘artistry’ component of nursing skill and competence which cannot be embodied in propositional form and is thus not simply taught or defined. Nursing practice does require its ‘novices’ to acquire skills and competencies but these alone do not define nursing and nursing practice. The list of skills identified through the documentary analysis could not then reflect nursing practice; a conceptual framework was required to ‘capture’ and ‘refine’ nursing’s skills. This will be explored within the next section of this paper on ‘Methods’.

## Methods

In order to deal with the skills in a structured way a theoretical/conceptual framework to ‘capture’ and organise the data meaningfully was developed; this facilitated the data extraction and avoided overly simplify the data into a list of tasks or skills. The complexity of nursing and nursing practice needed to be considered and captured in this process, hence illustrating the ‘work of the nurse’ in such a way that the members of the nursing profession could identify with the skills areas identified. Paradoxically peer reviewed literature has a significant number of papers (close to 300,000) on nursing work but the majority of these are concerned with three areas; workload and so numbers and skill mix of nursing staff in a variety of settings; specific clinical area activity such as perioperative practice, intensive care or other specialist settings and quite precise technical skills relate to those settings and lastly job satisfaction or strain, burnout or stress. On the other hand seminal works such as Lawler [13] explored the nature of nursing work from the perspective of the body. Allen [5] explored the notion of ‘boundaries’ in nursing as did Chiarella and Adrian [14]; this latter work explores the ethical issues related to bodily contact and the consequent possible intimacy and sexuality connotations. Earlier work from Allen [15] explored record keeping as a means of managing ‘routine nursing practice’—neither of these pragmatically explored what skills a nurse should be taught in a preparation for practice programme nor made any attempt to conceptualise nursing work or clinical practice.

Literature was explored that considered the work of the nurse; Anderson [16] explored the “terrain” and suggested that the work of the nurse needed to be ‘mapped’ (p.95); Utley-Smith (2004:166) [17] identified what she termed “factors”. These factors illustrated the ‘work of the nurse’ from Schwirian’s [18] work which identified six factors and 52 behaviours and skills with two dimensions—‘how often the nurse carries out the activity’ and secondly ‘how well does the nurse perform the activity?’ The’factors ranged from ‘direct care’ and ‘supervision’ through to ‘communication’ and ‘case load management’. These continue to be evident in nursing practice; they include items such as; ‘Develop a plan of care’; ‘Coordinate the plan of nursing care with the medical plan of care’ and ‘Perform technical procedures’ as well as aspects of ‘emotional care’ and ‘communication’. Meretoja, Erikson & Leino-Kilpi (2002:97) [19] outlined a number of indicators for nursing competence which they described as ‘domain categories’; these included ‘the helping role of the nurse’; ‘incorporating relevant research into practice’ and ‘administering medications safely’. These works provided the framework for the coding of the ‘skills lists’ gathered from the Australian universities delivering nursing programmes which will be explored later in this paper. The developed headings were Clinician, Communicator, Educator, Manager and Researcher.

### Design of the study

An inclusive methodology was employed with the intention of maximising contributions and ownership from the profession. All nursing schools in Australia were invited to provide data to support the research (*n* = 39). Firstly skills were audited and identified from a documentary analysis of nursing programme curriculum documents in Australia. Data extraction techniques were used to interpret and illicit meaning and gain understanding – this method is based on the work of Corbin & Strauss [20] and Rapley [21].

### Participants

The Heads/Deans of Nursing Schools in Australia were obtained through the Council of Deans of Nursing and Midwifery for Australia and New Zealand (CDNM-ANZ), they were invited to participate by identifying an individual within their school to act as a liaison with the researcher to provide curriculum documents and information on skills taught within their nursing programmes. The nominated individual, often a relatively junior member of the academic team within their organisation, provided the information requested in the form of curriculum documents and lists of skills taught.

### Data collection

Documents were requested to be submitted to the research team electronically so that the management and collation of the information could be performed readily. Non-responders were followed-up within one week by email and then if necessary an individual telephone call was made to explore issues and clarify requirements; thirty eight of the thirty nine schools of nursing responded by the end of the follow-up period. In the main, non–responders were relatively junior members of the academic staff team and sought clarification either with the Head of School or with the programme leader within their university.

### Data analysis

Documentary analysis is a recognised form of qualitative research [22]. The curriculum documents and skills lists submitted were reviewed by two independent parties. The “whole” of the data was initially reviewed to consider commonly taught skills e.g. monitoring vital signs were identified by all universities. A data extraction plan included listing and then organising and categorising the lists of skills submitted. This was undertaken using the conceptual framework derived from literature that explores the ‘work of the nurse’.

A body of literature explores theoretical frameworks for describing, and to a lesser extent conceptualising, nursing work. Anderson [16] explored the “terrain” and suggested that the work of the nurse needed to be ‘mapped’; Utley-Smith [17] identified what she termed “factors”. These factors illustrated the ‘work of the nurse’ from Schwirians [18] work which identified six factors and 52 behaviours and skills with two dimensions—‘how often the nurse carries out the activity’ and secondly ‘how well does the nurse perform the activity?’ The factors ranged from ‘direct care’ and ‘supervision’ through to ‘communication’ and ‘case load management’. These continue to be evident in nursing practice; they include items such as; ‘Develop a plan of care’; ‘Coordinate the plan of nursing care with the medical plan of care’ and ‘Perform technical procedures’ as well as aspects of ‘emotional care’ and ‘communication’. Meretoja, Erikson & Leino-Kilpi [19] outlined a number of indicators for nursing competence which they described as ‘domain categories’; these included ‘the helping role of the nurse’; ‘incorporating relevant research into practice’ and ‘administering medications safely’. These works provided the framework for the coding of the ‘skills lists’ gathered from the Australian universities delivering nursing programmes. The category headings used for coding were; Clinician, Communicator, Educator. Researcher and Manager; the data was then coded within these broad headings.

*Ethical Considerations:* a submission was made to the University of Wollongong’s Human Research Ethics Committee (HREC- HE08/142) in which all university Heads of Schools, participant groups, research areas and methods were listed. The documents for all aspects of the work were submitted; this included the participant information sheets and consent forms to all stakeholders, this included Heads of schools, clinical academics, students and assessors/supervisors. This was approved by the Human Research Ethics Committee. Rigorous annual reporting was completed to ensure that the researchers were adhering to ethical principles.

## Results and Discussion

### Responses

Initially 31 of the possible 39 universities responded to the initial written request to Heads of Schools for information relating to skills taught in eligibility to practice programmes in Australia. Initially four of those universities requested additional information and four did not respond. On providing additional information two universities submitted their skills taught and curriculum documents. Two other universities requested a discussion with the research team. Following this discussion these two respondents then submitted their universities documentation. The four non-responders were followed up initially be email and then by telephone and one respondent did not wish to participate. Of the documentation submitted a number appeared either incomplete or did not clearly indicate the skills taught within that particular programme. These participants were followed up by telephone or in person by the research team to clarify what information was required and to respond to any queries or questions. As a result of this process finally there were 38 out of 39 respondents who submitted curriculum documentation in an appropriate format.

### Initial analysis

Over 1300 skills were identified as being taught in nursing eligibility to practice preparation programmes across Australia. On removing repetitions this was reduced to around 1100, further analysis and combining brought this to 272 skills taught. The data was then coded using the conceptual framework; all 1300 skills were coded in to one of the five headings (i.e. clinician, researcher, etc.). These were then reviewed by the expert group and combined into a number of subsets under those headings—30 skills areas resulted; see Appendix: Table 3.

The initial listing of over 1300 skills was then revisited and recoded under the five headings see Table 1 below:

**Table 1:** Work of the nurse

	No of skills area	Universities	No of items
Clinician	13	38	1042
Communicator	4	38	61
Manager (Leader)	6	32	165
Researcher	2	18	17
Educator	5	20	32
Totals	30	–	1313

This table (Table 1) illustrates the five areas of ‘work of the nurse’ that were derived from the literature to represent clinical practice activities. One example will be explored as an illustration of the *Clinician* role of the practicing nurse’ the administration of *Medications and IV Products (*e.g. *safe and appropriate storage, administration and disposal of medications)* specifically.

Curriculum documents highlighted over 230 entries relating to ‘medication and IV products’ (see Table 2 below). The table illustrates the range of medication related activities—the frequently noted were ‘medications’; ‘medication/drug calculations’ and ‘IV medications including IVI’s’.

**Table 2:** Areas identified by universities related to medications

	Item	Number	
1	Eye dressing and ophthalmic medications	2	2
2	Legal responsibilities associated with medication administration	1	1
3	Medical orders on progress notes & medication chart	3	3
4	Medical, pharmacological, surgical, psychological and other treatments ~ respiratory, blood and	4	4
5	Medical orders on progress notes & medication chart	1	1
6	Anti-hypertensive medications	1	1
7	Ear & eye medication	4	4
8	First line medications	1	1
9	Intravenous Medication, including IVI’s	33	33
10	Medication calculation/Drug Calculations	26 + 13=	39
11	Medication	98	98
12	Paediatric medications	3	3
13	Psychotropic medications effects, management of	2	2
14	Rectal medications	1	1
15	Respiratory medications	2	2
16	S4D oral medication, administration	1 + 2=	3
17	SC Insulin	3	3
18	Sublingual – buccal	1	1
19	Topical Medications	1	1
20	5 R’s as a basic rule for drug admin	2	2
21	Cardiac emergency drugs, identifying	2	2
22	Describes main drugs	1	1
23	Psycho active/tropic drugs, administration and monitoring	2	2
24	Restricted drug management	1	1
25	PCA	13	13
26	Pain relief	1	1
	TOTAL		235

These skills taught in university programmes in Australia were identified along with twenty three other entries (see Table 2) relating to medication administration were located within the *Medications and IV Products* heading and placed in the Clinician category. In terms of the total skills identified there were 17.1 % relating to medication and IV administration in all clinical settings. Appendix: Table 4 illustrates all the 30 skills areas and the number of items’ within each category (total number 1376) and the number of universities (*n* = 38) who identified items taught within that skill area.

The four least identified areas taught within nursing programmes were within the ‘Educator’ sub heading. These included ‘acting as a resource’; ‘promotes self-care’; ‘demonstrates behaviour conducive to learning’; ‘learning and development culture’—universities only identified 16 skills taught in these four skills areas. A few examples relate to ‘continuing education’ and ‘identifying learning needs’ were included; other items such as ‘immunization’ and ‘health screening’ were listed.

A greater number (16) were noted which indicated aspects of education pertaining to the practitioner using ‘appropriate learning and teaching strategies in practice’ such as ‘patient teaching’, ‘patient education’ and ‘patient/family education’ as well ‘health education’ and ‘health promotion’. Parenting and child health promotions were identified here too, as well as some specific clinical care aspects such as managing diabetes or other medication compliance issues.

The 1300 (plus) individual skills refined in to the 30 skills areas reflect what is identified from university nursing programme curriculum documents in Australia; clearly a number of areas are highlighted by being mentioned multiple times in the documents from the responding universities. Most universities highlighted the need to teach students about assessing vital signs and about broader socio-political aspects of assessment in a variety of key clinical settings, namely acute tertiary hospitals, community and mental health care settings.

The data, through using an inclusive methodology, captured all aspects of teaching within the 38 nursing programmes. The rather broad range of skills is probably inevitable when the end product of nursing eligibility to practice programmes in Australia is a graduate who is eligible to practice in a wide range of clinical settings.

Accessing the data from universities was on the whole relatively straight forward however a number of universities had a range of individuals at different levels within their organisations, ‘overseeing’ or ‘managing’ these aspects of their courses. There was no clear or consistent approach; these individuals were often at the level of lecturer, usually with a level of responsibility in an individual subject/unit; or a person oversaw the ‘process’ that had a particular interest in skills teaching.

## Conclusions

This work reflects an audit of nursing skills taught in virtually all universities in Australia, it is a changing space; clearly curriculum content needs to change with both shifting demands of the public and changes in health technologies and care delivery.

National policy is shifting towards an approach that is philosophically seated within a health education framework in order to reduce the burden on the tertiary health care system however universities appear to be slow to change. The 5/6 yearly accreditation cycle clearly influences this ability to respond quickly to shifts in care delivery and health technology; developing and then accrediting takes two to three years and then the first graduates join industry three years later—understandably this cumulatively leads to a 5/6 year time delay.

Nursing work is changing and shifting to caring for clients and patients who are more acutely ill as in patients and so the focus for clients/patients in hospitals is towards more challenging morbidity and to avoiding mortality. In the community the shift is towards caring for individuals at home and so reducing the tertiary care in all settings mental health as well as acute care.

## Appendix

**Table 3:** Skills areas identified following the documentary analysis of university curriculae

*Skill Area*
1. Planning of nursing care (e.g. range of varied settings/client’s needs)
2. Understanding the different roles of RNs in different treatment or care settings (e.g. aged care, rural and remote, acute, mental health, etc.)
3. Medications and IV products (e.g. safe and appropriate administration of medications)
4. Clinical monitoring and management - Use of assessment tools (e.g. Haemodynamic/respiratory assessment, MMSE, RUDAS, etc.) all forms of assessment are included here.
5. Technology and Informatics (e.g. IVI management systems, patient information systems, etc)
6. Personal care – ability to assess, plan, implement and evaluate care of clients across a range of settings using a holistic, comprehensive nursing model
7. Mental health nursing care (e.g. application of assessment tools and care strategies and interventions)
8. Knowledge of key nursing implications of common medical/surgical patient presentations
9. Clinical interventions – preparing, assisting after care (investigations/surgery/diagnostic)
10. Professional nursing behaviours –includes collaborative approaches to care (e.g. advocacy, scope of practice, being aware of one’s self, etc.)
11. Privacy and dignity (e.g. culturally acceptable practice, personal space, respectful)
12. Dealing with emotional and bereaved people (e.g. breaking bad news, dealing with anger, etc.)
13. Dementia related skills (e.g. behavioural and psychosocial symptoms of dementia).
14. Coordinating skills regarding nursing process – uses a range of appropriate assessment strategies and skills across a range of settings.
15. Leadership skills.
16. Preventing risk and promoting safety – duty of care (e.g. strategies for reducing risk, risk assessment, etc. – promoting self-care)
17. Case manager (e.g. coordination of care, crisis/emergency situation management, etc.).
18. Teamwork and multidisciplinary team working.
19. Supervisory skills.
20. Cultural competence (e.g. cross-cultural care, culturally safe and appropriate practice).
21. Therapeutic nursing behaviours/respectful of personal space.
22. Efficient and effective communication (e.g. with professionals in other disciplines).
23. Communication and documentation i.e. verbal including handovers and non-verbal including documentation.
24. Learner/evidence based practitioner (e.g. appropriate application of practice evidence).
25. Critical analysis and reflective thinking (e.g. use of reflection and critical incidents, evidence of linking theory to practice).
26. Demonstrates teaching/educator skills (e.g. utilising appropriate teaching and learning strategies in practice).
27. Acts as a resource.
28. Promotes self-care (e.g. specific gender and lifespan related information and strategies).
29. Demonstrates behaviour conducive to learning (e.g. approachable and supportive).
30. Learning and developmental culture – learning environment (e.g. relates to an environment conducive to learning and personal and professional growth as a new graduate).

**Table 4:** Table illustrating from highest to lowest the skills ‘***items***’ taught in nursing programmes in Australia

*Skill Area*	*Skill Grouping*	*Universities* (*n = 38*)	Items (*n* = 1376)
3. Medications and IV products (e.g. safe and appropriate administration of medications)	Clinician	38	235
2. Understanding the different roles of RNs in different treatment or care settings (e.g. aged care, rural and remote, acute, mental health, etc)	Clinician	37	206
4. Clinical monitoring and management - Use of assessment tools (e.g. Haemodynamic/respiratory assessment, MMSE, RUDAS, etc.) all forms of assessment are included here.	Clinician	38	205
9. Clinical interventions – preparing, assisting after care (investigations/surgery/diagnostic)	Clinician	32	100
16. Preventing risk and promoting safety – duty of care (e.g. strategies for reducing risk, risk assessment, etc. – promoting self-care)	Manager	34	99
6. Personal care – ability to assess, plan, implement and evaluate care of clients across a range of settings using a holistic, comprehensive nursing model	Clinician	38	96
10. Professional nursing behaviours - includes collaborative approaches to care (e.g. advocacy, scope of practice, being aware of one’s self, etc.)	Clinician	32	66
7. Mental health nursing care (e.g. application of assessment tools and care strategies and interventions)	Clinician	31	61
12. Dealing with emotional and bereaved people (e.g. breaking bad news, dealing with anger, etc.)	Clinician	22	35
23. Communication and documentation i.e. verbal including handovers and non-verbal including documentation.	Communicator	32	28
8. Knowledge of key nursing implications of common medical/surgical patient presentations	Clinician	23	26
11. Privacy and dignity (e.g. culturally acceptable practice, personal space, respectful)	Clinician	14	26
14. Coordinating skills regarding nursing process – uses a range of appropriate assessment strategies and skills across a range of settings.	Manager	24	26
5. Technology and Informatics (e.g. IVI Management systems, patient information systems, etc.)	Clinician	29	23
1. Planning of nursing care (e.g. range of varied settings/client’s needs)	Clinician	29	22
17. Case manager (e.g. coordination of care, crisis/emergency situation management, etc.).	Manager	18	22
21. Therapeutic nursing behaviours/respectful of personal space.	Communicator	28	21
26. Demonstrates teaching/educator skills (e.g. utilising appropriate teaching and learning strategies in practice).	Educator	20	16
25. Critical analysis and reflective thinking (e.g. use of reflection and critical incidents, evidence of linking theory to practice).	Researcher	13	9
15. Leadership skills.	Manager	4	8
22. Efficient and effective communication (e.g. with professionals in other disciplines).	Communicator	3	8
18. Teamwork and multidisciplinary team working.	Manager	13	6
13. Dementia related skills (e.g. behavioural and psychosocial symptoms of dementia).	Clinician	4	4
19. Supervisory skills.	Manager	1	4
20. Cultural competence (e.g. cross-cultural care, culturally safe and appropriate practice).	Communicator	4	4
24. Learner/evidence based practitioner (e.g. appropriate application of practice evidence).	Researcher	4	4
27. Acts as a resource.	Educator	4	4
28. Promotes self-care (e.g. specific gender and lifespan related information and strategies).	Educator	7	4
29. Demonstrates behaviour conducive to learning (e.g. approachable and supportive).	Educator	5	4
30. Learning and developmental culture – learning environment (e.g. relates to an environment conducive to learning and personal and professional growth as a new graduate).	Educator	5	4

## Abbreviations

ALTC: Australian Learning and Teaching Council
ANMC: Australian Nursing and Midwifery Council
ANMAC: Australian Nursing and Midwifery Accreditation Council
CDNM: Council of Deans of Nursing and Midwifery
I.V. or I.V.I.: Intravenous Infusion
MMSE: Mini Mental State Examination
NMBA: Nursing and Midwifery Board of Australia
OLT: Office of Teaching and Learning
RN: Registered Nurse
RUDAS: Rowland Universal Dementia Assessment Scale
